# The Dfam community resource of transposable element families, sequence models, and genome annotations

**DOI:** 10.1186/s13100-020-00230-y

**Published:** 2021-01-12

**Authors:** Jessica Storer, Robert Hubley, Jeb Rosen, Travis J. Wheeler, Arian F. Smit

**Affiliations:** 1 Institute for Systems Biology, Seattle, WA 98109 USA; 2 University of Montana, Missoula, MT 59812 USA

## Abstract

Dfam is an open access database of repetitive DNA families, sequence models, and genome annotations. The 3.0–3.3 releases of Dfam (https://dfam.org) represent an evolution from a proof-of-principle collection of transposable element families in model organisms into a community resource for a broad range of species, and for both curated and uncurated datasets. In addition, releases since Dfam 3.0 provide auxiliary consensus sequence models, transposable element protein alignments, and a formalized classification system to support the growing diversity of organisms represented in the resource. The latest release includes 266,740 new de novo generated transposable element families from 336 species contributed by the EBI. This expansion demonstrates the utility of many of Dfam’s new features and provides insight into the long term challenges ahead for improving de novo generated transposable element datasets.

**Supplementary Information:**

The online version contains supplementary material available at 10.1186/s13100-020-00230-y.

## Introduction

Significant portions of many genomes are composed of transposable element (TE) copies. TE-derived sequence decays in the genome over time, making its discovery and characterization challenging. However, accurate annotation and description of these elements is crucial in understanding their impact on the genome in which they reside and the evolution of a species as a whole. The influence of TEs on the genome and/or species can be direct, such as insertions into coding regions, exaptation to new functions, or chromosomal rearrangements as a consequence of non-homologous recombination, or indirect, as with an “arms race” between the host and resident parasite. TE instances have long been identified in genomes through a combination of two complementary strategies: de novo detection, and database-driven annotation. In de novo detection, a variety of methods are used to recognize and categorize remnants of TE activity. The families identified in this fashion are typically further curated and cataloged in databases such as Dfam. In database-driven annotation each sequence in that database is aligned to the genome being annotated, with the best-scoring alignment determining the label of the genomic sequence. Such databases have long used consensus sequences to represent each family. However, such searches tend to miss highly-diverged sequences, prompting us to explore the utility of profile methods [1, 2] to increase sensitivity.

In 2012, we released Dfam [3], a database of TE families from the human genome in which each family was represented by a multiple sequence alignment (MSA) and a profile hidden Markov model (HMM). Profile HMMs [4, 5] yield sensitivity gains in part by modeling the position-specific residue and indel (insertion and deletion) variability found in family MSAs. The first release of Dfam was based on the design of similar databases of protein (Pfam) and RNA (Rfam) families [6, 7]. In addition to improving annotation sensitivity through the use of profile HMMs, Dfam demonstrated decreased false discovery rates through rigorously defined thresholds [8]. An additional advantage of these databases is the preservation of a multiple sequence alignment of representative family members, the seed alignment. The seed alignment is model-agnostic, provides details on coverage and fragmentation, and supplies essential provenance for the family.

Subsequent releases of Dfam refined the prototype database and modestly expanded the curated libraries to five model organisms (4150 families). In 2018, Dfam received funding to move from a proof-of-principle to a production community resource by (1) scaling the system architecture, (2) supporting multiple model types (HMMs and consensus sequences) derived from the seed alignment, (3) improving annotation speed and quality, and most importantly (4) engaging the community in its further development. In this paper we will describe accomplishments represented in the latest release series (Dfam 3.0 through 3.3) and the challenges that lay ahead. These accomplishments include support for consensus models, a hierarchical TE classification system with an interactive explorer, a TE termini library encompassing various DNA transposon classes, and a framework for “uncurated” or “raw” data sets in Dfam alongside the existing curated data sets.

Dfam 3.3 currently houses 273,655 families: 112,455 retrotransposons, 101,711 DNA transposons, and 59,489 other repeats that include interspersed repeats of unknown origin, satellite regions and/or other non-TE entries to avoid annotating non-coding RNA genes as TEs. At present, Dfam’s coverage of organismal diversity is smaller than that of Repbase, owing to Repbase being a closed database and having two decades longer to accumulate data; with the development of an open framework for community contribution, the pace of data acquisition in Dfam is expected to close that gap quickly.

## Consensus models

While the use of HMMs allows for improved detection of TE copies in genomes, most sequence analysis algorithms (Smith-Waterman, Needleman-Wunsch etc.) and popular sequence analysis tools (BLAST, BLAT etc.) act directly on string representations of sequences (e.g. consensus sequences). Likewise, programs used to define new TEs (de-novo repeat finders), to extend fragmented models, to unravel the relationship of related TEs, to classify elements or to describe biological features like exons are not typically able to generate or take advantage of HMMs directly. Each TE model in Dfam therefore should be accompanied by a simple sequence model; a consensus sequence derived from the seed alignment is the logical candidate.

The use of a consensus sequence as a first-order model for sequence families has a rich history of demonstrated utility [2, 9–11]. A consensus is typically made by considering the occupancy and composition of columns in a multiple sequence alignment of TE copies. A basic consensus caller might assign for any given column the majority nucleotide found in the column regardless of occupancy (the number of homologous nucleotides in the column vs the number of gaps). A more sophisticated caller would account for gaps and make base calls reflecting the observed rates of substitutions in the given genome.

**Fig. 1:**
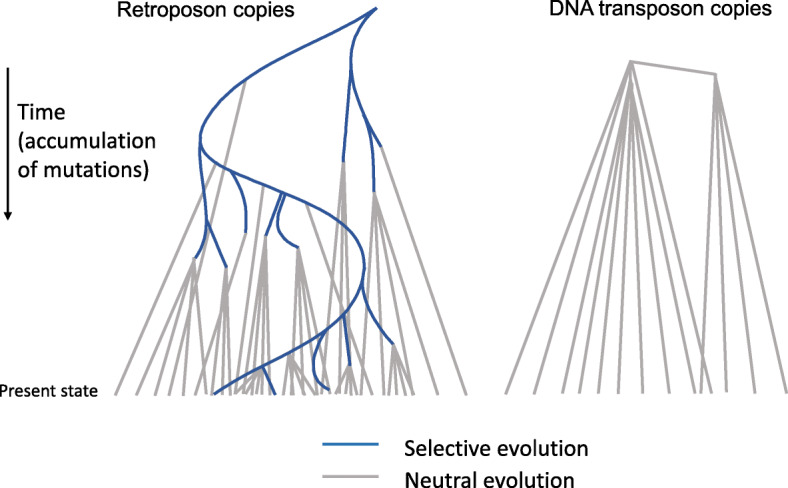
Typical phylogenetic structure of retroposon and DNA transposon families. After multiple mutations have occurred in the evolving class I TE, the relative ordering of copies may be distinguished by these changes as they cosegregate. The presence of such clusters or “subfamilies” of TE copies is a good indication that they arose via retrotransposition

Most TE-derived sequences are under no functional constraint and accumulate mutations in a random and neutral fashion. Given this random noise, an informed consensus for a sufficient number of properly aligned copies may be expected to reproduce the original active TE sequence. This bears out particularly well for most “class II elements” or “DNA transposons” in eukaryotes; due to the trans-activity of the transposase on the genomic copies, these do not tend to evolve during their short life in a genome, and thus create copies that have a star-like phylogenetic relationship to the original sequence (Fig. 1) [12]. The situation is more complicated for most class I elements, which duplicate via a reverse transcription step; thanks to the cis-activity of the reverse transcriptase on its own mRNA, they may evolve, e.g. to escape the host’s defense mechanisms, and propagate in a genome for hundreds of millions of years. For these elements, careful clustering of copies into so-called subfamilies will result in a series of interrelated consensus sequences that can be interpreted as snapshots of the TE sequence during its evolution, though each may still be a composition of divergent active elements.

The concept that consensus sequences approach the original TE sequence has been demonstrated by the resurrection of recently extinct TEs through modification of a dead copy to the consensus [13, 14] and by the recovery of expected sequence features for ancient elements. For example, the consensus sequences of many coding TEs that were active > 100 MYA in our genome contain full-length ORFs [15], despite the fact that their individual copies have accumulated so many mutations that they on average share less than half the original nucleotides with each other and often cannot be pairwise aligned.

While Repbase was initiated as a reference database containing (single copy) prototypes of genomic interspersed repetitive DNA [16], we began developing consensus sequence representations by 1994 [11]. Not only did this endeavor explain the biological origin of most repetitive DNA, the use of consensus sequences rather than genomic copies improved the detection of older and therefore more diverged copies as well. A collection of genomic copies has redundancy and contains low complexity sequences like simple repeats expanded in individual copies, both of which result in lower specificity. More importantly, individual copies of a TE are separated by, on average, twice as many mutations as they are from the original sequence approached by a consensus, improving sensitivity dramatically.

In Dfam we now provide both profile HMM and auxiliary consensus sequence models for each family in the database. Both are derived from a single seed alignment, allowing for provenance to be maintained and both models to be simultaneously improved. Most importantly, Dfam maintains a correspondence between consensus and HMM positions so that alignments produced by either may be compared directly. In Dfam, consensus sequences are generated for each family using a caller that we originally employed to build many of the consensus sequences for Repbase. It assigns the base with the highest score using a log-odds substitution matrix that reflects neutral substitution patterns in a genome, including e.g. the strong GC- > AT bias in mammals. It also infers ancestral CpG sites by accounting for the frequency of TG and CA dimers in neighboring columns [17].

## Classification system

**Fig. 2:**
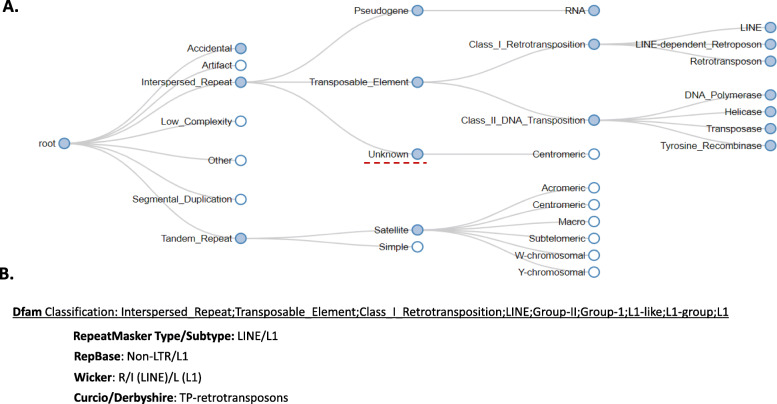
Dfam TE classification system and visualization tool. **a** A small portion of the Dfam TE classification tree depicted using the dynamic visualization found at the Dfam website. Filled in circles represent internal nodes of the tree while hollow circles are leaf nodes in the classification tree. A classification is specified by concatenating the path through the classification tree. For example, the classification “Interspersed_Repeat;Unknown” is highlighted in the tree. **b** In addition, wherever possible a mapping is provided between classification systems. The Dfam classification for the L1 group of LINEs is shown with the equivalent classifications in several other systems

Without classification, a TE library is of limited use. While entries in Dfam have always been classified, in this release series we developed a new classification system for repetitive sequences in eukaryotic genomes. In addition, an interactive tool was developed for the website to assist in exploring the new system in the form of an identification key (Fig. 2).

Classification of TEs poses specific problems that may prevent a universal solution to be found [18]. A purely cladistic approach is impossible as TEs are polyphyletic (they have many independent origins) and because their relationship is reticulated (sections of TEs can have entirely different evolutionary histories, due to recombinations, gene captures and nested insertions). Classic SINEs, which have originated many times from fortuitous positioning of an internal promoter (e.g. in a small RNA gene) and the 3′ fragment of an active LINE [15, 19] provide an example for both these issues. Nevertheless, most currently-used classification systems for eukaryotic TEs are very similar and are based on hybrids of cladistic, mechanistic, and structural approaches.

In 1989, David Finnegan introduced an early classification with just four classes [20]. His basic division between TEs that transpose via an RNA intermediate (class I) and those that “transpose directly from DNA to DNA” (class II) is still used by most. Considering the fundamental impact of the trans-activity of class II proteins on their transcripts and the cis -activity of class I proteins on their genomic copies, this is indeed a primary division (Fig. 1). At the time, very few types of eukaryotic TEs were known, and his further divisions of class I elements into those with and without long terminal repeats (LTRs), and divisions of class II elements into those with short and long terminal inverted repeats (TIRs) has not survived the onslaught of new data, although LTR and non-LTR (LINE) elements still form valid clades, at least from the reverse-transcriptase point of view [21].

When we introduced RepeatMasker in 1995, we needed a succinct classification to fit in the slightly modified cross_match format [22] that we used to annotate genomic DNA. We chose a three-level form coded as “level1/level2-level3” (e.g. “DNA/hAT-Charlie”). We adopted Finnegan’s LTR, LINE and class II (“DNA”) divisions and added SINE and a number of non-TE classes for the first divisions, the three class I elements reflecting a bias towards the frequency of elements encountered in the human and other mammalian genomes. Second and third divisions represent clades of elements based on reverse transcriptase (RT) or transposases phylogenies. Non-autonomous elements whose movement depends upon the coding capacity of autonomous elements, were grouped within the autonomous elements’ classification, based on similarities of the LTRs or TIRs in the absence of any coding sequence. Entries in Repbase more or less inherited this simple classification hierarchy. In later years, attempts were made to reflect as much as possible of the classification in the name of the elements [23]. The classification system suggested in 2007 by researchers with a primarily plant genomics background [24] has the same basis in Finnegan and follows a similar logic; in order to display compact classification on an annotation line, they suggested a three-letter class-order-superfamily code to add to each “family” classification. The “subfamily” was suggested to be used in the TE’s name itself.

Our classification, like those before, combines a mechanistic, cladistic and structural approach. Where possible, the relationship of the RT in class I elements and transposase, helicase, or DNA polymerase in class II elements guides the tree. While non-autonomous LTR elements tend to remain dependent on the autonomous element from which they formed and can be classified with these, LINE-dependent non-autonomous elements have a variety of origins. They are separated by those with a small RNA derived pol III internal promoter (the SINEs) and other elements. The latter category is a grab bag of sorts, classified by the type of LINE they depend upon, and contains elements mostly consisting of LINE-material to hodgepodges like SVA [25]. The modular, classic SINEs are organized by their 5′ small RNA-derived, core, and 3′ LINE-derived modules. Class II elements are divided in the four fundamental mechanisms of propagation so far known in eukaryotes, “cut-and-paste” via a linear or circular dsDNA, “rolling circle”, and “self-synthesizing” groups, after which the phylogenetic relationship of the transposase, recombinase, helicase, or DNA polymerase, respectively, takes over. Like non-autonomous LTR elements, most non-autonomous DNA transposons can be classified based on their TIR combined with their target site duplication (TSD) pattern. We therefore do not provide structural categories like LARD (large LTR retrotransposon derivatives) or MITE (miniature inverted–repeat transposable elements).

The Dfam classification system does not display a ranked hierarchy as there will never be satisfying definitions for what a class, order, family or subfamily of TEs constitutes, while with the addition of new elements and growing knowledge of their relationship, the number of branches, and therefore subdivisions, along some parts of the tree will remain in flux (see Supplementary Figure 1 and Table S1). Wicker et al. proposed to define a family as a group of TEs that can be aligned over at least 80 bp and show 80% + identity covering 80% or more of the alignment [24]. Meant as a pragmatic definition, it has been pointed out that applying it would lead “to an unpredictable mix of monophyletic, paraphyletic and polyphyletic groups” [26]. Strictly following this rule will also not be practical, as, for example, newly identified TEs intermediate between known families will force these to be merged over time and the aforementioned reticulate relationship of TEs could join radically different TEs in one family. Also, some of the ranks are already in use for other purposes: the term “family” is often used for any group of aligned TE copies for which a consensus or HMM has been derived and, in animal TE annotation, “subfamilies” either indicate subsets of class I TE copies that share multiple co-segregating differences from the rest (Fig. 1) or sets of particular internal deletion products of an autonomous class II transposon. With its lack of taxonomic ranks, our schema avoids these issues.

TEs may also be classified by their transposition mechanism and classification systems based on the mechanism of integration and chemistry of the transposition reaction have been proposed [27, 28]. These have the benefit of being able to integrate the wide variety of TEs active in prokaryotes, but are somewhat hampered by the lack of knowledge on the details of transposition by new, bioinformatically discovered TEs. Furthermore, written specifically to include prokaryotic TEs, the mechanistic classifications do not have the fundamental division in cis-active and trans-active elements, brought about by the separation of transcription and translation in eukaryotes. While the focus of the RepeatMasker/Repbase/Wicker classification on eukaryotes and on reverse transcriptase phylogeny has been criticized [29], a unified eukaryotic/prokaryotic TE classification would be unwieldy. In the future, we will explore the use of an independent classification for prokaryotic TEs.

A TE family can be classified as belonging to any node in the classification tree by concatenating the names along the path from the root to the designated node. For example, the highlighted node in Fig. 2 is referenced with the string “Interspersed_repeat;Unknown”. This enables partial classifications to be made and node labels to be reused. All classifications are linked to the corresponding RepeatMasker, Repbase, Wicker-et-al. or Curcio-Derbyshire classification, where they are available.

While most interspersed repeats identified by de novo repeat finding programs are derived from TEs, alternative origins include (i) simple tandem repeats, originating independently at many sites, (ii) long tandem repeats like satellites, found at multiple (sub)telomeric and centromeric sites, (iii) segmental duplications, (iv) common coding motifs like zinc fingers, and (v) gene families. In mammals, the most common non-TE source of interspersed repeats are retro(pseudo)genes that have been accidently copied by the LINE1-mechanism; some small structural RNAs occur with over a thousand copies [30]. While our classification system includes these categories for annotation purposes, most of these entries should not be part of Dfam. Satellites and small structural RNAs are included in Dfam, but shorter tandem repeats are better detected by specialized programs like TRF [31] and ULTRA [32] and the inclusion of segmental duplications, cellular transcripts or coding regions would lead to much false annotation.

## Transposon termini

Most methods to categorize newly identified TEs are pipelines that rely heavily on finding homology to existing classified TEs [17, 33–35]. When a curated library of a related species exists, near-full-length matches at the DNA level are often found that allow proper class assignment. A translated comparison to a TE protein database can classify many models with (remnants of) coding sequence. However, due to the recombinant and modular tendencies of TEs, sequence homology is not always sufficient evidence for classification, especially if similarities are fragmentary or weak. For example, many non-autonomous class I and II elements carry insertions or fragments of non-related TEs, while a model matching the 3′ end of a LINE element may represent a SINE instead. Even functional TE coding regions can be misleading as related proteins have been repurposed in disparate TEs; for example, the transposases of the cut-and-paste Ginger transposons are closely related to the integrases of both Gypsy retrotransposons and Maverick/polinton self-synthesizing elements [36, 37]. Needless to say, TEs without (remnant) coding sequence or related entries in the database will remain unclassified using this method alone.

There are fortunately other characteristics and sequence features of TEs that can confirm the mechanism by which the element propagated, and in turn, how it should be classified. Clusters of copies with co-segregating mutations generally imply that the TE is a class I element, and, given enough copies, their complete absence suggests a class II element. A simple-repeat tail and TSDs of variable length indicate movement via target-primed reverse transcription, which requires the protein products of LINEs. When models have TG…CA termini and carry a poly-adenylation signal, while their copies are flanked by 4–6 bp TSDs, they likely represent solitary LTRs, which, through homologous recombination, can vastly outnumber complete LTR elements in a genome.

In our experience, many models that resist automatic categorization represent non-autonomous class II elements that actually can be classified in detail based on the pattern of the terminal 20–50 bp. This is particularly true for the ubiquitous TIR transposons: their transposases specifically recognize and bind the terminal sequences, to the point that these are sufficient for an element to retain or obtain mobility [38, 39]. As a result, transposons with similar transposases (the basis of their classification) have similar termini. While these terminal homologies are too short to appear significant in a whole database search, they can be found by comparing the 30–60 bp termini of new elements to the 30–60 bp termini of all classified class II elements and filtering the output by orientation and position. Characteristic TSDs can cement classification. Over the years, we classified thousands of short sequences in Repbase this way, including ancient elements that were active in the common ancestor of all amniotes.

**Fig. 3:**
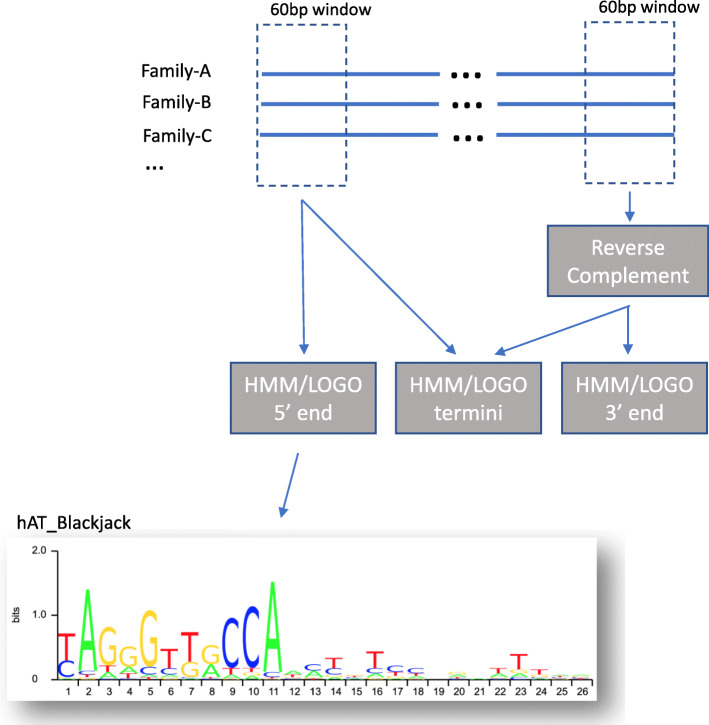
Generation of HMMs and sequence LOGOs for DNA transposon termini. The first/last 60 bp of family consensi belonging to a single classification of DNA Transposons are piled up and aligned (without gaps) by hand. Profile HMMs are developed for each end and for the combination of the two to determine if a stronger signal may be obtained in that fashion. Finally, LOGOs are generated for each HMM and displayed on the Dfam website

In Dfam, we now provide terminal sequence signatures for 64 categories of class II elements, for use in classifying new TE models. To create these signatures, we lined up the 5′ and 3′ 60 bp of all members of a particular type that seem to have clearly defined ends (e.g. as indicated by the presence of the expected TSDs, Fig. 3). Minor modifications were made to some of the nearly 12,000 remaining consensus sequences in order to have each start at the true beginning or end of the TE. This most commonly involved removing (partial) target site duplications or adding one or more Ns when comparison to others showed the sequence to be short. The alignments are ungapped, however, and it is possible that more signal can be obtained by allowing a few indels. We used HMMER (hmmbuild) to develop HMMs for the 5′ ends, the 3′ ends, and, in case of TIRs, the combined termini.

The LOGOs of the termini can be viewed on the “Classifications” page on the Dfam website, and are organized by class II subclasses (e.g., Crypton, Helitron, TIR, etc.). This allows for easy visualization of the base conservation at each position in the terminal sequences and comparisons between the 5′ and 3′ termini. The full set of profiles may be downloaded and will be updated as new elements are added to each class.

## Dfam growth

The development of a curated TE library for a given species is a specialized and mostly manual task that has only incrementally improved since the inception of TE databases. We recognize that as reference genome sequencing increases at a faster rate, and until automated curation methods improve, uncurated datasets (mostly de novo generated TE libraries) will far outpace the development of curated libraries. There are some advantages to making uncurated datasets available through a resource such as Dfam: (1) they can be used as simple genome masking libraries, and the fragmentation and redundancy that are the hallmark of datasets derived from de novo discovery tools are not detrimental in that context, (2) cataloging uncurated families provides a shared starting point for community curation efforts, and (3) these datasets will provide a resource for developing per-family and per-library quality metrics as well as improved automated curation tools.

**Fig. 4:**
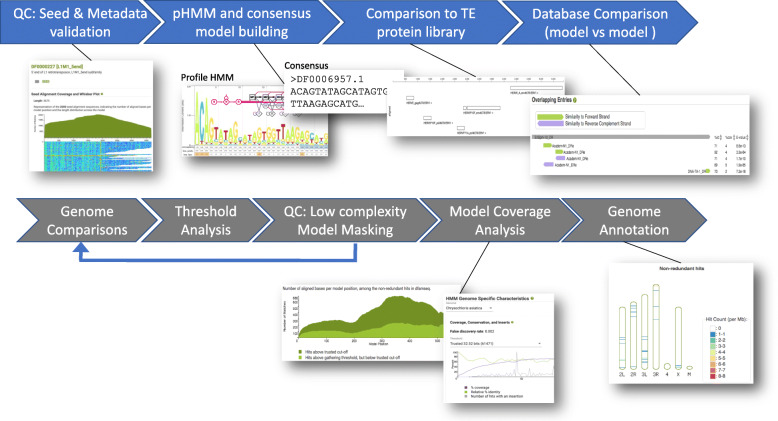
Dfam analysis pipeline. The full Dfam analysis pipeline consists of a set of sequential analysis steps depicted above with examples of the products produced. For uncurated families only the first portion of the pipeline (colored in blue) is initially conducted

In the latest release of Dfam we have added support for uncurated datasets, denoting these families using the new accession prefix “DR” and limiting/altering the analysis and metadata displayed for these families (Fig. 4). For instance, a DR family has both a consensus and a profile HMM generated for it, but does not have rigorously characterized false discovery rate thresholds as for curated families [3] due to the lack of pre-calculated assembly annotations. However, uncurated families do contain provenance for the seed alignment, standard metadata (description, classification, taxa, citations etc), TE protein matches, relationships with other families, and model details. By limiting the analysis for DR families to those that facilitate future curation efforts, we can scale Dfam to handle this growing data category and provide early access to newly discovered TE families.

**Fig. 5:**
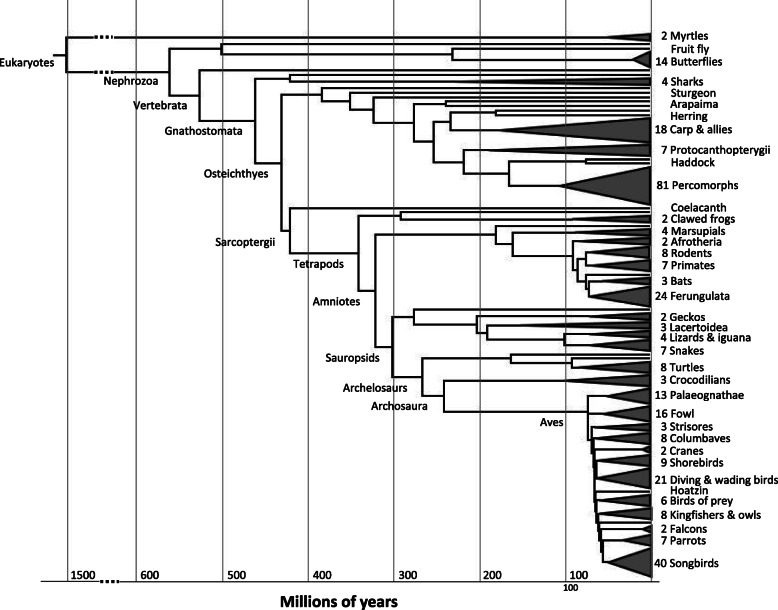
The phylogenetic distribution of species in Dfam release 3.3. A subset of species are named and species-rich groups are collapsed into triangles, with the number of species indicated. Major branch points have been labeled according to the NCBI taxonomy. Branching times and order of the tree are roughly following that of the OneZoom project [40] with modifications based on more specific research; for example, the branching order within the birds is according to Prum et al. [41]

To demonstrate the new features in the latest release of Dfam and the challenges/opportunities this type of dataset will create, we imported RepeatModeler results generated by the EBI on 336 assemblies (Fig. 5). This import of 266,740 families dwarfed the existing Dfam by 40-fold. The seed alignments typically produced by RepeatModeler were not available from these runs, therefore we used the RepeatModeler consensus libraries to generate new seed alignments from annotated instances identified by RepeatMasker. By using a library-based approach rather than individual family searches, we avoided assigning TE instances to more than one family. This step was followed by an iterative extension process (manuscript in preparation) that is based upon an approach used in RepeatScout [42] to further extend fragmented families.

Due to the scale of the dataset, only minimal prefiltering of the families was performed before import. Families with > 80% tandemly repetitive sequence and less than 100 bp of contiguous non tandemly repetitive sequence (according to TRF) were removed. This filtered 894 families. TRF was run with a maximum period of 20 bp to preserve common and complex satellite sequences in the dataset.

The number of TE models in Dfam has grown from 6915 to 273,655, largely because of the RepeatModeler libraries for 336 additional species contributed by the EBI. Table S2 contains statistics for these EBI additions alone. 116 bony fish, 133 bird and 47 mammalian species form the bulk of the additions, and numbers for these clades alone are displayed in the table. A generally higher variety of recognizable TEs in fish and lower variety in birds is reflected in the data: bony fish (35% of the new species) contributed 58.4% of the added models, while mammals and birds (14 and 40% of the new species) each contributed about 9.3% of the new models (Table S2). The remaining 10% of the EBI dataset, and therefore 23% of the TE diversity, belong to other groups outside of bony fish, mammals, and birds that include: plants, cartilaginous fish, turtles and lizards.

An analysis of the TE classes and superfamilies in each of the aforementioned clades depicted that bony fish contribute a large number of models across class II TE superfamilies, and are consequently enriched for class II TEs (Supplementary Figure 2 and Table S2). Birds and mammals contribute to and are enriched for LINEs (CR1 and L1, respectively), in addition to endogenous retrovirus (ERV)-like LTR elements.The EBI dataset confirms large-scale patterns visible in addition to providing data regarding TE expansion for individual organisms.

**Fig. 6:**
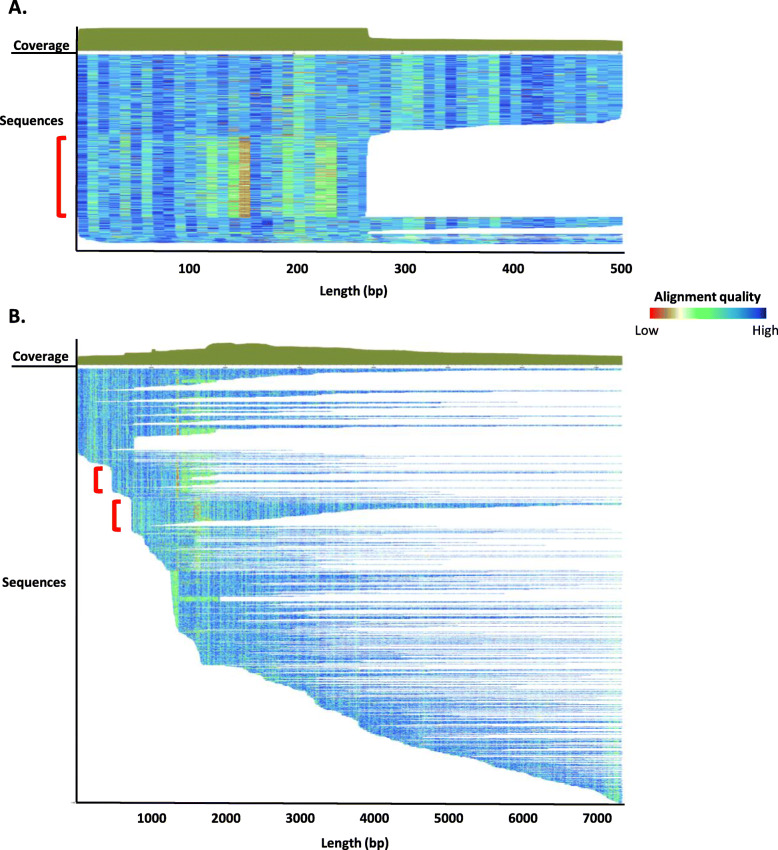
Seed alignment examples from raw Dfam 3.3 entries. A) ERV1 (LTR) (DR0086957.1; *Eulemur macaco* (black lemur)). The red bracket indicates a group of sequences differing in length and divergence patterns. 506 sequences contribute to the seed alignment for this 503 bp model. B) L1 sequence (DR0215804.1; *Phyllostomus discolor* (pale spear-nosed bat)). The red brackets indicate two groups of sequences that differ in their 5′ alignment. One thousand one hundred eighty-six sequences contribute to the seed alignment for this 7353 bp model. Each sequence is represented by a single row (sorted by start position) where the color gradient indicates alignment quality (red = low; blue = high) over 10 bp non-overlapping windows. The solid green shape over the two seed alignments indicate the coverage along the model consensus sequence. Note: the two panels differ in scale

Examples of the seed alignments generated as part of the aforementioned pipeline can be observed in Fig. 6. The seed alignment for any model provides researchers with a wealth of information including sequence coverage, length, and number of sequences contributing to the model. From a curator’s perspective, information like divergence patterns and blocks of seemingly truncated sequences are also evident. For example, a block of sequences in an LTR alignment that show a different divergence pattern and/or appears to be truncated at the same position relative to the consensus sequence are likely to indicate a subfamily structure within the model (Fig. 6a, red box). This alignment pattern reflects the biology of the TE, as LTR sequences regularly recombine their 5′ and 3′ ends to form new families [43]. Similarly, blocks of outwardly truncated sequences close to the 5′ end in an L1 seed alignment reflect a tendency for differing patterns present in the 5′ UTR regions [44] (Fig. 6b). Closer inspection of these sequence blocks within the alignment is warranted to determine if a subfamily structure is present.

By providing the RepeatModeler output for the additional 336 species, Dfam encourages the TE community to lend their expertise, thus improving this dataset so that it moves from a raw dataset to a highly curated one. We are working on providing tools for the community to curate and improve upon these and existing curated datasets.

## Architectural and Interface improvements

The architecture of Dfam was refactored in Dfam 3.0 to prepare the resource for housing TE families, seed alignments, and sequence models at scale. The Dfam website (https://www.dfam.org) has been updated to provide both (i) a front end intended for human interaction through a web browser (the ‘portal’) and (ii) an API served over HTTP to support programmatic access to Dfam data. The source code for both projects is available on GitHub (https://github.com/Dfam-consortium/) and released under the CC0 public domain dedication. The API uses computer-friendly data formats such as JSON and tab-separated values (as appropriate), which makes it more suitable as a data source for community-developed tools than the human-oriented format of the website.

The portal has gained several significant new features. To support the massive scale of family data we replaced the old one-page-per-letter approach to family organization with a “Browse” page that supports sorting and filtering on multiple criteria such as name, classification, taxon, or keywords. Some similar filters have also been added to the “Relationships” tab to restrict results to related species. The “Features” tab has been added to the per-family page to display curated features (binding sites, hand-curated coding sequences etc), as well as blastx matches against other known TE protein sequences.

The architectural changes also facilitated the merger of Dfam and Dfam_consensus [45], creating a single resource for both consensus and HMM sequence models for each family. During the merger, Dfam inherited the seed alignment visualization from Dfam_consensus. This visualization shows both coverage (how many representatives cover each portion of the alignment) and the conservation patterns within the alignment, shown as a heatmap indicating how closely each member aligns to the consensus (as seen in Fig. 6).

Dfam also now includes an authentication system and public data submission system; this is the first step in a planned development of a curation workbench that will provide users with tools to edit and curate data uploaded by themselves or (eventually) others.

## Software/tool distribution improvements

One of the main hurdles with using bioinformatic pipelines and tools is the complexity of the installation and configuration necessary to use them. Software containers are an increasingly popular way to tackle this problem, by delivering an executable user environment that comes with pre-installed and pre-configured software packages. In order to support community curation efforts we have developed containers for Docker and Singularity, housing pre-installed versions of our latest tools (e.g. RepeatMasker, RepeatModeler, Coseg, RMBlast) and dependencies as well as external open-source tools (e.g. HMMER, mafft, cdhit, TRF). More information along with instructions for use are available at https://github.com/Dfam-consortium/TETools/. As part of our outreach efforts, we will be eliciting recommendations for including additional packages in future releases.

Notably these containers include FamDB, a new HDF5-based Dfam export format and associated query tools for offline access to Dfam. FamDB files contain family consensi/HMMs and the NCBI Taxonomy data related to these families in a format that allows for fast offline access from the command line. The current release of FamDB includes all Dfam consensus sequences, HMMs, metadata, and 61,003 taxa from NCBI’s taxonomy database [46] related to these families. Lookups for information on a single taxon or family complete in about a second; extraction of consensus sequences (FASTA, EMBL) or HMMs for all TE families found in Human (including ancestral repeats) complete in about 3 to 4 s. Due to indexing, the run time for data queries is largely independent of the total number of TEs in the database: it takes about the same amount of time to extract the human library from a FamDB file including only the curated subset of Dfam (6915 entries) as for the full database (273,655).

## Future challenges/directions

The curation of multi-species TE databases involves many manual tasks (e.g. defragmentation, classification validation, lineage assignment) and many subjective decisions (e.g. redundancy removal, pseudogene removal, subfamily characterization) that have not been universally standardized into protocols. With the growth of uncurated TE libraries and their inclusion in databases such as Dfam, it has become necessary to develop protocols for many of these tasks and to consider the challenges introduced by this data growth. Here, we discuss two of these topics that we will be focusing on in the near term.

### Subfamilies

When a family is believed to exhibit subfamily structure, there are a variety of methods available to cluster the family copies into subfamily groups. A subfamily structure is suspected when there is a wide divergence distribution of family members, or directly observing distinct groups of co-segregating subsequences in a seed alignment. Wicker used the aforementioned 80/80/80 rule to cluster sequences into subfamilies: sequences are clustered such that all members of the subfamily share at least 80% sequence identity and at least 80% sequence coverage with length greater than 80 bp. A strategy to define subfamilies was used in the analysis of bread wheat subgenomes by applying a 90/90 or 95/95 rule in which 90 or 95% sequence identity and 90 or 95% sequence coverage was used to cluster sequences into subfamilies [47, 48]. However, further testing should be completed to determine if this type of threshold accurately splits sequences into subfamilies of all TE types across a wide array of species.

The methods used to develop subfamily models vary widely, from the manual clustering of copies in a multiple sequence alignment by eye, to automated clustering algorithms [49], and network-based approaches [50]. Wildly different subfamily sets can be produced by these alternative methods, or even by using slightly different parameterizations within one method. The large number of fine-grained subfamilies produced by some of these methods is not of practical use for identifying copies of the superfamily in a genome or specifically labeling individual copies confidently [51] with current sequence similarity search algorithms. Still, in aggregate the tree structure and subfamily membership are valuable datasets for studying family evolution and databases can play a role in the standardisation of this data.

**Fig. 7:**
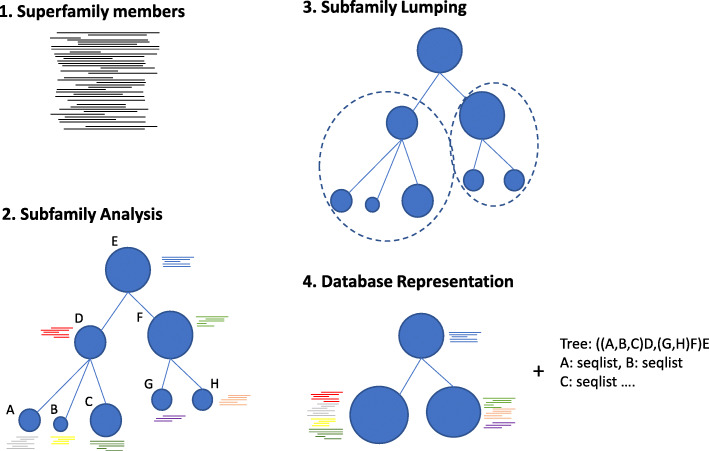
Database subfamily representation. Proposed database representation for TE subfamilies maintaining a detailed phylogenetic structure while reducing the representative models for practical genome-scale annotation. The TE seed alignment (1) from a family with evidence of subfamily structure is analysed by a clustering method to produce a detailed subfamily structure and membership (2). Sequence models are developed for subfamilies and lumped (3) if model performance isn’t improved by the subdivision of two or more subfamilies. The lumped families and their corresponding seed alignments are added to the database (4) with metadata holding the detailed tree structure and seed sequence membership for each subfamily

For future releases of Dfam we will explore ways to set a minimum sequence distance threshold for inclusion in Dfam as a subfamily. The threshold should reflect the current sensitivity and specificity of both HMM and consensus based search algorithms and act on the detailed subfamily tree to cluster closely-related subfamilies (lumping their copies together). The original detailed tree structure and individual copy membership need not be lost (Fig. 7), but stored alongside the superfamily as a combination of newick data and fine-scale seed alignment sequence subfamily labels for further study and use.

### Redundancy/fragmentation detection

**Fig. 8:**
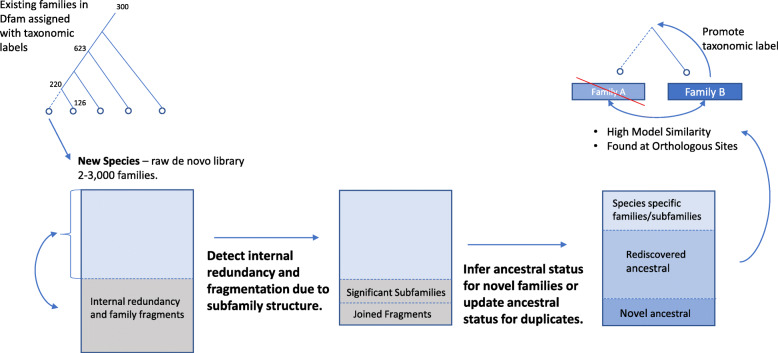
Redundancy/Fragmentation removal challenges. Both inter- and intra-library redundancy is present in de novo datasets and are currently resolved through manual curation. Interlibrary redundancy is often the result of unresolved subfamily structure (e.g. internal deletion products of DNA transposons) that confounds discovery and produces both redundant and fragmented families. Intra library redundancy is an inherent aspect analyzing a single species in isolation. For each new species these ancestral families need to be resolved by comparison to existing families, and by considering presence at orthologous sites

Ideally, a TE database should contain a single full-length entry for each transposing family. Unfortunately that overly simple definition doesn’t account for the fine detail of subfamily expansions, recombinations, deletion products, and mosaicism exhibited by many TE families. These processes lead to necessary redundancy in a library. Another form of redundancy, that is not desirable, is the direct result of (1) re-detection of ancestral families in the de novo analysis of two or more related species, (2) the confounding effects of sequence variation on de novo detection methods leading to rediscovery, and (3) inadequate clustering in pipelines that run multiple discovery methods and merge the results (Fig. 8). In addition, differences in the representative set of TE copies, in the alignment parameters, and the selection of model building parameters will lead to subtle differences between models generated for the same family; making automatic redundancy detection difficult.

Through the expansion of the Dfam database via the addition of diverse sets of species and their associated TEs, it will become necessary to detect redundancy automatically. One approach would be to use a comparative genomics approach to assess TE insertions at orthologous sites to resolve interspecies redundancies while improving the taxonomic labels for each family (Fig. 8).

Fragmentation is an additional problem apparent in most de novo datasets. In some cases this directly relates to the structure of the observed TE copies appearing as distinct patterns within a genome (e.g. full length LTRs with internal sequence, and solo LTRs) or coverage bias in a families copies (e.g 5′ end of LINE families). In either case joining these fragments into a complete family is the desired result. Fragmentation is often identified during manual curation as a family fragment is extended and subsequently matched to another fragment in the library over the extended region. Another approach would be to use genome annotations for the uncurated library to identify significant collinearity among family pairs and automatically group families together.

## Conclusion

The new Dfam release has expanded the number and scope of species included in the database, allowing for enhanced genome annotation while fostering the development of highly curated TE libraries for use in research. In addition, a unified eukaryotic TE classification scheme and HMMs for DNA transposon termini now on Dfam, provide additional details for researchers to utilize in their TE research. Combined with an expanded TE library, the new database architecture, improved interfaces, and simplified software distribution, Dfam offers a collaborative platform for the TE research community. Collaborative efforts and increased datasets will be necessary to tackle problems such as those mentioned above: subfamily identification, library redundancy and fragmentation. We invite the TE research community to provide feedback on the challenges discussed here and to join us in these efforts to further Dfam development.

## Supplementary Information

**Additional file 1.**

**Additional file 2.**

**Additional file 3.**

**Additional file 4: Table S2.** EBI dataset TE class and superfamily contribution and enrichment by clade for Supplemental Figure 2.

## Data Availability

The datasets generated during and/or analyzed during the current study are available at the Dfam portal: https://dfam.org. Software generated for this project is located in the Dfam consortium repository: https://github.com/Dfam-consortium.
