# Supplementary material for: The Dfam community resource of transposable element families, sequence models, and genome annotations
Source: Mob DNA. 2021 Jan 12;12:2. doi: 10.1186/s13100-020-00230-y (PMC7805219; doi:10.1186/s13100-020-00230-y)
Supplement: Supplementary file 1 — Additional file 1. [file 13100_2020_230_MOESM1_ESM.pdf]

Supplemental Figure 2. The full Dfam classification system displayed as a linear tree with node descriptions where available.

```
root
├─Accidental
│ │   Virus families that are more likely the
│ │   result of spontaneous integration rather
│ │   than Retrotranposition.
│ │
│ └─Normally_Non-integrating_Virus
│       Virus families that are more likely the
│       result of spontaneous integration rather
│       than Retrotranposition.
│
├─Artifact
│     Elements often found in cloning vectors
│     that may contaminate sequencing
│     projects.
│
├─Interspersed_Repeat
│ │   Primarily or only found in centromeric
│ │   regions.
│ │
│ ├─Pseudogene
│ │ │   Copies of a transfer RNA (tRNA) gene or
│ │ │   pseudogene.
│ │ │
│ │ └─RNA
│ │   │   Copies of a transfer RNA (tRNA) gene or
│ │   │   pseudogene.
│ │   │
│ │   ├─rRNA
│ │   │     Copies of a ribosomal RNA (rRNA) gene or
│ │   │     pseudogene.
│ │   │
│ │   ├─scRNA
│ │   ├─snRNA
│ │   │     Copies of a small nuclear RNA (snRNA)
│ │   │     gene or pseudogene.
│ │   │
│ │   └─tRNA
│ │         Copies of a transfer RNA (tRNA) gene or
│ │         pseudogene.
│ │
│ ├─Transposable_Element
│ │ │   A placeholder for not-yet-classified
│ │ │   non-autonomous LTR elements with short
│ │ │   internal sequences.
│ │ │
│ │ ├─Class_II_DNA_Transposition
│ │ │ │   Widespread class of hAT elements,
│ │ │ │   including the Arthur and Zaphod elements
│ │ │ │   in ancient mammals.
│ │ │ │
│ │ │ ├─DNA_Polymerase
│ │ │ │ │   Archaeal and bacterial mobile elements
│ │ │ │ │   at the origin of CRISPR-Cas immunity.
│ │ │ │ │
│ │ │ │ ├─Casposon
│ │ │ │ │     Archaeal and bacterial mobile elements
│ │ │ │ │     at the origin of CRISPR-Cas immunity.
│ │ │ │ │
│ │ │ │ └─Maverick
│ │ │ │   └─Maverick-Mavirus
│ │ │ │
```

```
│ │ │ ├─Helicase
│ │ │ │ │   Circular ssDNA Intermediate.
│ │ │ │ │
│ │ │ │ ├─Helitron-1
│ │ │ │ └─Helitron-2
│ │ │ ├─Transposase
│ │ │ │ │   Widespread class of hAT elements,
│ │ │ │ │   including the Arthur and Zaphod elements
│ │ │ │ │   in ancient mammals.
│ │ │ │ │
│ │ │ │ ├─Academ
│ │ │ │ │ │   Family of Academ transposons described
│ │ │ │ │ │   in rust fungi.
│ │ │ │ │ │
│ │ │ │ │ ├─Academ-1
│ │ │ │ │ │     First described family of Academ DNA
│ │ │ │ │ │     transposon.
│ │ │ │ │ │
│ │ │ │ │ ├─Academ-2
│ │ │ │ │ │     Second described family of Academ DNA
│ │ │ │ │ │     transposon.
│ │ │ │ │ │
│ │ │ │ │ └─Academ-H
│ │ │ │ │         Family of Academ transposons described
│ │ │ │ │         in rust fungi.
│ │ │ │ │
│ │ │ │ ├─CACTA
│ │ │ │ │ │   Group of DNA transposons at the basis of
│ │ │ │ │ │   our adaptive immune system.
│ │ │ │ │ │
│ │ │ │ │ ├─CMC
│ │ │ │ │ │ │   So-far animal-specific elements with a
│ │ │ │ │ │ │   specific DDE transposase.
│ │ │ │ │ │ │
│ │ │ │ │ │ ├─Chapaev_group
│ │ │ │ │ │ │ │   So-far animal-specific elements with a
│ │ │ │ │ │ │ │   specific DDE transposase.
│ │ │ │ │ │ │ │
│ │ │ │ │ │ │ ├─Chapaev
│ │ │ │ │ │ │ │     So-far animal-specific elements with a
│ │ │ │ │ │ │ │     specific DDE transposase.
│ │ │ │ │ │ │ │
│ │ │ │ │ │ │ └─Chapaev-3
│ │ │ │ │ │ │         So-far animal-specific elements with a
│ │ │ │ │ │ │         specific DDE transposase.
│ │ │ │ │ │ │
│ │ │ │ │ │ ├─EnSpm
│ │ │ │ │ │ └─Mirage
│ │ │ │ │ └─Transib
│ │ │ │ │         Group of DNA transposons at the basis of
│ │ │ │ │         our adaptive immune system.
│ │ │ │ │
│ │ │ │ ├─Dada
│ │ │ │ ├─Ginger
│ │ │ │ │ ├─Ginger-1
│ │ │ │ │ └─Ginger-2
│ │ │ │ ├─IS3EU
│ │ │ │ ├─Kolobok
│ │ │ │ │ ├─Hydra-specific_Branch
│ │ │ │ │ ├─Kolobok-E
│ │ │ │ │ ├─Kolobok-H
│ │ │ │ │ └─T2
│ │ │ │ ├─Merlin
│ │ │ │ ├─Mutator-like
│ │ │ │ │ │   Mutator-like Element aka MULE.
```

```
│   │   │   │   │   │
│   │   │   │   │   ├─F
│   │   │   │   │   ├─MuDR
│   │   │   │   │   ├─NOF
│   │   │   │   │   └─Ricksha
│   │   │   │   ├─Novosib
│   │   │   │   ├─PIF-Harbinger
│   │   │   │   │ ├─HarbS
│   │   │   │   │ ├─Harbinger
│   │   │   │   │ ├─ISL2EU
│   │   │   │   │ └─Spy
│   │   │   │   ├─P_Element
│   │   │   │   │ └─Fungi-specific_Branch
│   │   │   │   ├─PiggyBac_Group
│   │   │   │   │ ├─PiggyBac
│   │   │   │   │ ├─PiggyBac-A
│   │   │   │   │ └─PiggyBac-X
│   │   │   │   ├─Sola
│   │   │   │   │ ├─Sola-1
│   │   │   │   │ ├─Sola-2
│   │   │   │   │ └─Sola-3
│   │   │   │   ├─Tc1-Mariner
│   │   │   │   │ │   So far animal-specific mariner-like
│   │   │   │   │ │   elements with the mammalian Tigger1 as
│   │   │   │   │ │   type specimen.
│   │   │   │   │ │
│   │   │   │   │ ├─Ant1
│   │   │   │   │ │     A group of DNA transposons so far
│   │   │   │   │ │     limited to fungi.
│   │   │   │   │ │
│   │   │   │   │ ├─Cweed
│   │   │   │   │ ├─Gizmo
│   │   │   │   │ ├─IS885
│   │   │   │   │ ├─ISRm11
│   │   │   │   │ ├─Mariner
│   │   │   │   │ ├─Mogwai
│   │   │   │   │ ├─Sagan
│   │   │   │   │ ├─Stowaway
│   │   │   │   │ ├─Tc1
│   │   │   │   │ ├─Tc2-group
│   │   │   │   │ │ │   So far animal-specific mariner-like
│   │   │   │   │ │ │   elements with the mammalian Tigger1 as
│   │   │   │   │ │ │   type specimen.
│   │   │   │   │ │ │
│   │   │   │   │ │ ├─Fot1
│   │   │   │   │ │ ├─Pogo
│   │   │   │   │ │ ├─Tc2
│   │   │   │   │ │ └─Tigger
│   │   │   │   │ │       So far animal-specific mariner-like
│   │   │   │   │ │       elements with the mammalian Tigger1 as
│   │   │   │   │ │       type specimen.
│   │   │   │   │ │
│   │   │   │   │ ├─Tc4
│   │   │   │   │ └─m44
│   │   │   │   ├─Zator
│   │   │   │   ├─Zisupton
│   │   │   │   └─hAT
│   │   │   │     │   Widespread class of hAT elements,
│   │   │   │     │   including the Arthur and Zaphod elements
│   │   │   │     │   in ancient mammals.
│   │   │   │     │
│   │   │   │     ├─Activator
│   │   │   │     │     A large class in plants and animals
│   │   │   │     │     named after the maize element.
│   │   │   │     │
```

```
│ │ │ │   ├─Blackjack
│ │ │ │   │     A group of hAT transposons possibly
│ │ │ │   │     closest to Charlie elements.
│ │ │ │   │
│ │ │ │   ├─Charlie
│ │ │ │   │     Mostly animal-specific hAT transposons
│ │ │ │   │     with the mammalian Charlie1 as type
│ │ │ │   │     specimen.
│ │ │ │   │
│ │ │ │   ├─Pegasus
│ │ │ │   ├─Restless
│ │ │ │   ├─Tag1
│ │ │ │   ├─Tip100
│ │ │ │   │     Widespread class of hAT elements,
│ │ │ │   │     including the Arthur and Zaphod elements
│ │ │ │   │     in ancient mammals.
│ │ │ │   │
│ │ │ │   ├─hAT1
│ │ │ │   ├─hAT19
│ │ │ │   ├─hAT5
│ │ │ │   ├─hAT6
│ │ │ │   ├─hATm
│ │ │ │   ├─hATw
│ │ │ │   ├─hATx
│ │ │ │   └─hobo
│ │ │ └─Tyrosine_Recombinase
│ │ │     └─Crypton
│ │ │       ├─Crypton-A
│ │ │       ├─Crypton-C
│ │ │       ├─Crypton-F
│ │ │       ├─Crypton-H
│ │ │       ├─Crypton-I
│ │ │       ├─Crypton-R
│ │ │       ├─Crypton-S
│ │ │       ├─Crypton-V
│ │ │       └─Crypton-X
│ │ └─Class_I_Retrotransposition
│ │   │   A placeholder for not-yet-classified
│ │   │   non-autonomous LTR elements with short
│ │   │   internal sequences.
│ │   │
│ │   ├─LINE
│ │   │ │   A subgoup of RTE elements with the
│ │   │ │   bovine BovB as the type specimen.
│ │   │ │
│ │   │ ├─Group-I
│ │   │ │ │   A group of LINE  so far specific to
│ │   │ │ │   Diatoms.
│ │   │ │ │
│ │   │ │ ├─Ambal
│ │   │ │ │     A group of LINE  so far specific to
│ │   │ │ │     Diatoms.
│ │   │ │ │
│ │   │ │ ├─CRE
│ │   │ │ │ ├─CRE-1
│ │   │ │ │ └─CRE-2
│ │   │ │ └─Odin
│ │   │ └─Group-II
│ │   │   │   A subgoup of RTE elements with the
│ │   │   │   bovine BovB as the type specimen.
│ │   │   │
│ │   │   ├─Genie
│ │   │   ├─Group-1
│ │   │   │ │   Containing the sister clades of
│ │   │   │ │   Deceiver, Dong-R4, and Dualen LINE
```

```
│ │   │   │ │   elements.
│ │   │   │ │
│ │   │   │ ├─L1-like
│ │   │   │ │ │   A large group of LINE elements with
│ │   │   │ │ │   mammalian LINE-1 (L1) as type specimen.
│ │   │   │ │ │
│ │   │   │ │ ├─DRE
│ │   │   │ │ ├─L1-group
│ │   │   │ │ │ │   A large group of LINE elements with
│ │   │   │ │ │ │   mammalian LINE-1 (L1) as type specimen.
│ │   │   │ │ │ │
│ │   │   │ │ │ ├─L1
│ │   │   │ │ │ │     A large group of LINE elements with
│ │   │   │ │ │ │     mammalian LINE-1 (L1) as type specimen.
│ │   │   │ │ │ │
│ │   │   │ │ │ └─Tx1
│ │   │   │ │ └─Zorro
│ │   │   │ ├─Proto-1
│ │   │   │ ├─R2-like
│ │   │   │ │ │   Containing the sister clades of R2, Hero
│ │   │   │ │ │   and NeSL LINEs.
│ │   │   │ │ │
│ │   │   │ │ ├─Hero
│ │   │   │ │ ├─NeSL
│ │   │   │ │ └─R2
│ │   │   │ └─R4-like
│ │   │   │   │   Containing the sister clades of
│ │   │   │   │   Deceiver, Dong-R4, and Dualen LINE
│ │   │   │   │   elements.
│ │   │   │   │
│ │   │   │   ├─Deceiver
│ │   │   │   ├─Dong-R4
│ │   │   │   └─Dualen
│ │   │   └─Group-2
│ │   │     │   A subgoup of RTE elements with the
│ │   │     │   bovine BovB as the type specimen.
│ │   │     │
│ │   │     ├─Proto-2
│ │   │     ├─R1-like
│ │   │     │ │   Containing the closely related LOA and
│ │   │     │ │   R1 LINEs.
│ │   │     │ │
│ │   │     │ ├─CR1-group
│ │   │     │ │ │   A large group of LINE elements with
│ │   │     │ │ │   mammalian LINE-2 (L2) as type specimen.
│ │   │     │ │ │
│ │   │     │ │ ├─CR1
│ │   │     │ │ │ │   A large group of LINE elements with
│ │   │     │ │ │ │   Chicken Repeat 1 (CR1) as type specimen.
│ │   │     │ │ │ │
│ │   │     │ │ │ └─Zenon
│ │   │     │ │ ├─L2-group
│ │   │     │ │ │ │   A large group of LINE elements with
│ │   │     │ │ │ │   mammalian LINE-2 (L2) as type specimen.
│ │   │     │ │ │ │
│ │   │     │ │ │ └─L2
│ │   │     │ │ │       A large group of LINE elements with
│ │   │     │ │ │       mammalian LINE-2 (L2) as type specimen.
│ │   │     │ │ │
│ │   │     │ │ └─Rex-Babar
│ │   │     │ ├─R1-group
│ │   │     │ │ │   Containing the closely related LOA and
│ │   │     │ │ │   R1 LINEs.
│ │   │     │ │ │
│ │   │     │ │ ├─I-group
```

```
│ │   │     │ │ │ │   Containing the sister classes of I and
│ │   │     │ │ │ │   Jockey LINE elements.
│ │   │     │ │ │ │
│ │   │     │ │ │ ├─I
│ │   │     │ │ │ └─Jockey
│ │   │     │ │ └─R1-subgroup
│ │   │     │ │   │   Containing the closely related LOA and
│ │   │     │ │   │   R1 LINEs.
│ │   │     │ │   │
│ │   │     │ │   ├─LOA
│ │   │     │ │   └─R1
│ │   │     │ └─Tad1
│ │   │     └─RTE-like
│ │   │       │   A subgoup of RTE elements with the
│ │   │       │   bovine BovB as the type specimen.
│ │   │       │
│ │   │       ├─ORTE
│ │   │       └─RTE-group
│ │   │         │   A subgoup of RTE elements with the
│ │   │         │   bovine BovB as the type specimen.
│ │   │         │
│ │   │         ├─BovB
│ │   │         │     A subgoup of RTE elements with the
│ │   │         │     bovine BovB as the type specimen.
│ │   │         │
│ │   │         ├─RTE
│ │   │         └─RTE-X
│ │   ├─LINE-dependent_Retroposon
│ │   │ │   SINEs lacking a central region or with
│ │   │ │   central region yet unrelated to that of
│ │   │ │   other SINEs.
│ │   │ │
│ │   │ ├─Lacking_Small_RNA_pol_III_Promoter
│ │   │ │ │   Nonautonomous retroposons derived from
│ │   │ │ │   RTE-type LINEs.
│ │   │ │ │
│ │   │ │ ├─I-derived
│ │   │ │ │     Nonautonomous retroposons derived from
│ │   │ │ │     I-type LINEs.
│ │   │ │ │
│ │   │ │ ├─L1-dependent
│ │   │ │ │ │   A retroposon mobilized by an L1-type
│ │   │ │ │ │   LINE.
│ │   │ │ │ │
│ │   │ │ │ └─SVA
│ │   │ │ ├─L1-derived
│ │   │ │ │     Nonautonomous retroposons derived from
│ │   │ │ │     L1-type LINEs.
│ │   │ │ │
│ │   │ │ ├─L2-derived
│ │   │ │ │     Nonautonomous retroposons derived from
│ │   │ │ │     L2-type LINEs.
│ │   │ │ │
│ │   │ │ ├─R4-derived
│ │   │ │ │     Nonautonomous retroposons derived from
│ │   │ │ │     R4-type LINEs.
│ │   │ │ │
│ │   │ │ └─RTE-derived
│ │   │ │       Nonautonomous retroposons derived from
│ │   │ │       RTE-type LINEs.
│ │   │ │
│ │   │ └─SINE
│ │   │   │   SINEs lacking a central region or with
│ │   │   │   central region yet unrelated to that of
│ │   │   │   other SINEs.
```

```
│ │   │   │
│ │   │   ├─5S-RNA_Promoter
│ │   │   │ │   SINE with a 3' end derived from an RTE-
│ │   │   │ │   type LINE.
│ │   │   │ │
│ │   │   │ ├─Deu-core
│ │   │   │ │ │   SINE with a 3' end derived from a
│ │   │   │ │ │   L2-type LINE.
│ │   │   │ │ │
│ │   │   │ │ ├─L2-end
│ │   │   │ │ │     SINE with a 3' end derived from a
│ │   │   │ │ │     L2-type LINE.
│ │   │   │ │ │
│ │   │   │ │ └─Unknown_LINE-dependent
│ │   │   │ ├─MIR-core
│ │   │   │ │ │   SINE with a 3' end derived from an RTE-
│ │   │   │ │ │   type LINE.
│ │   │   │ │ │
│ │   │   │ │ └─RTE-end
│ │   │   │ │       SINE with a 3' end derived from an RTE-
│ │   │   │ │       type LINE.
│ │   │   │ │
│ │   │   │ ├─No_or_Unknown_Core
│ │   │   │ │ │   SINE with a 3' end derived from an RTE-
│ │   │   │ │ │   type LINE.
│ │   │   │ │ │
│ │   │   │ │ └─RTE-end
│ │   │   │ │       SINE with a 3' end derived from an RTE-
│ │   │   │ │       type LINE.
│ │   │   │ │
│ │   │   │ └─Sauria-core
│ │   │   │   │   SINE with a 3' end derived from an RTE-
│ │   │   │   │   type LINE.
│ │   │   │   │
│ │   │   │   └─RTE-end
│ │   │   │         SINE with a 3' end derived from an RTE-
│ │   │   │         type LINE.
│ │   │   │
│ │   │   ├─7SL-RNA_Promoter
│ │   │   │ │   A SINE family specific to primates and
│ │   │   │ │   rodents.
│ │   │   │ │
│ │   │   │ └─No-core
│ │   │   │   │   A SINE family specific to primates and
│ │   │   │   │   rodents.
│ │   │   │   │
│ │   │   │   └─L1-dependent
│ │   │   │     │   A SINE family specific to primates and
│ │   │   │     │   rodents.
│ │   │   │     │
│ │   │   │     └─Alu
│ │   │   │           A SINE family specific to primates and
│ │   │   │           rodents.
│ │   │   │
│ │   │   ├─U-RNA_Promoter
│ │   │   │ │   SINEs lacking a central region or with
│ │   │   │ │   central region yet unrelated to that of
│ │   │   │ │   other SINEs.
│ │   │   │ │
│ │   │   │ └─No_or_Unknown_Core
│ │   │   │   │   SINEs lacking a central region or with
│ │   │   │   │   central region yet unrelated to that of
│ │   │   │   │   other SINEs.
│ │   │   │   │
│ │   │   │   └─Unknown_LINE-dependent
```

```
│ │   │   ├─Unknown_Promoter
│ │   │   │ │   SINE with a 3' end derived from an RTE-
│ │   │   │ │   type LINE.
│ │   │   │ │
│ │   │   │ ├─Ceph-core
│ │   │   │ │ │   SINE with a 3' end derived from an RTE-
│ │   │   │ │ │   type LINE.
│ │   │   │ │ │
│ │   │   │ │ └─RTE-end
│ │   │   │ │       SINE with a 3' end derived from an RTE-
│ │   │   │ │       type LINE.
│ │   │   │ │
│ │   │   │ └─MIR-core
│ │   │   │   │   SINE with a 3' end derived from an RTE-
│ │   │   │   │   type LINE.
│ │   │   │   │
│ │   │   │   ├─RTE-end
│ │   │   │   │     SINE with a 3' end derived from an RTE-
│ │   │   │   │     type LINE.
│ │   │   │   │
│ │   │   │   └─Unknown_LINE-dependent
│ │   │   ├─tRNA_Promoter
│ │   │   │ │   SINE with a 3' end derived from a
│ │   │   │ │   L2-type LINE.
│ │   │   │ │
│ │   │   │ ├─Ceph-core
│ │   │   │ │ │   SINE with a 3' end derived from an RTE-
│ │   │   │ │ │   type LINE.
│ │   │   │ │ │
│ │   │   │ │ ├─RTE-end
│ │   │   │ │ │     SINE with a 3' end derived from an RTE-
│ │   │   │ │ │     type LINE.
│ │   │   │ │ │
│ │   │   │ │ └─Unknown_LINE-dependent
│ │   │   │ ├─Deu-core
│ │   │   │ │ │   SINE with a 3' end derived from an RTE-
│ │   │   │ │ │   type LINE.
│ │   │   │ │ │
│ │   │   │ │ ├─CR1-end
│ │   │   │ │ │     SINE with a 3' end derived from a
│ │   │   │ │ │     CR1-type LINE.
│ │   │   │ │ │
│ │   │   │ │ ├─I-end
│ │   │   │ │ │     SINE with a 3' end derived from a I-type
│ │   │   │ │ │     LINE.
│ │   │   │ │ │
│ │   │   │ │ ├─L2-end
│ │   │   │ │ │     SINE with a 3' end derived from a
│ │   │   │ │ │     L2-type LINE.
│ │   │   │ │ │
│ │   │   │ │ ├─RTE-end
│ │   │   │ │ │     SINE with a 3' end derived from an RTE-
│ │   │   │ │ │     type LINE.
│ │   │   │ │ │
│ │   │   │ │ └─Unknown_LINE-dependent
│ │   │   │ ├─MIR-core
│ │   │   │ │ │   SINE with a 3' end derived from an RTE-
│ │   │   │ │ │   type LINE.
│ │   │   │ │ │
│ │   │   │ │ ├─L2-end
│ │   │   │ │ │     SINE with a 3' end derived from a
│ │   │   │ │ │     L2-type LINE.
│ │   │   │ │ │
│ │   │   │ │ ├─Mermaid
│ │   │   │ │ ├─RTE-end
```

```
│ │   │   │ │ │     SINE with a 3' end derived from an RTE-
│ │   │   │ │ │     type LINE.
│ │   │   │ │ │
│ │   │   │ │ └─Unknown_LINE-dependent
│ │   │   │ ├─Meta-core
│ │   │   │ │ │   Animal-wide conserved SINE central
│ │   │   │ │ │   region.
│ │   │   │ │ │
│ │   │   │ │ └─Unknown_LINE-dependent
│ │   │   │ ├─No-core
│ │   │   │ │ │   A rodent specific SINE family.
│ │   │   │ │ │
│ │   │   │ │ └─L1-dependent
│ │   │   │ │   │   A rodent specific SINE family.
│ │   │   │ │   │
│ │   │   │ │   └─B2
│ │   │   │ │         A rodent specific SINE family.
│ │   │   │ │
│ │   │   │ ├─No_or_Unknown_Core
│ │   │   │ │ │   SINE with a 3' end derived from a
│ │   │   │ │ │   Tad1-type LINE.
│ │   │   │ │ │
│ │   │   │ │ ├─BovB-end
│ │   │   │ │ │     SINE with a 3' end derived from a BovB-
│ │   │   │ │ │     type LINE.
│ │   │   │ │ │
│ │   │   │ │ ├─CR1-end
│ │   │   │ │ │     SINE with a 3' end derived from a
│ │   │   │ │ │     CR1-type LINE.
│ │   │   │ │ │
│ │   │   │ │ ├─I-end
│ │   │   │ │ │     SINE with a 3' end derived from a I-type
│ │   │   │ │ │     LINE.
│ │   │   │ │ │
│ │   │   │ │ ├─Jockey-end
│ │   │   │ │ ├─L1-dependent
│ │   │   │ │ │     A retroposon mobilized by an L1-type
│ │   │   │ │ │     LINE.
│ │   │   │ │ │
│ │   │   │ │ ├─L2-end
│ │   │   │ │ │     SINE with a 3' end derived from a
│ │   │   │ │ │     L2-type LINE.
│ │   │   │ │ │
│ │   │   │ │ ├─R1-end
│ │   │   │ │ │     SINE with a 3' end derived from an
│ │   │   │ │ │     R1-type LINE.
│ │   │   │ │ │
│ │   │   │ │ ├─R2-end
│ │   │   │ │ │     SINE with a 3' end derived from an
│ │   │   │ │ │     R2-type LINE.
│ │   │   │ │ │
│ │   │   │ │ ├─RTE-end
│ │   │   │ │ │     SINE with a 3' end derived from an RTE-
│ │   │   │ │ │     type LINE.
│ │   │   │ │ │
│ │   │   │ │ ├─Rex-end
│ │   │   │ │ │     SINE with a 3' end derived from an Rex-
│ │   │   │ │ │     type LINE.
│ │   │   │ │ │
│ │   │   │ │ ├─Tad1_End
│ │   │   │ │ │     SINE with a 3' end derived from a
│ │   │   │ │ │     Tad1-type LINE.
│ │   │   │ │ │
│ │   │   │ │ └─Unknown_LINE-dependent
│ │   │   │ ├─Sauria-core
```

```
│ │   │ │ │   SINE with a 3' end derived from an RTE-
│ │   │ │ │   type LINE.
│ │   │ │ │
│ │   │ │ ├─L2-end
│ │   │ │ │     SINE with a 3' end derived from a
│ │   │ │ │     L2-type LINE.
│ │   │ │ │
│ │   │ │ ├─RTE-end
│ │   │ │ │     SINE with a 3' end derived from an RTE-
│ │   │ │ │     type LINE.
│ │   │ │ │
│ │   │ │ └─Unknown_LINE-dependent
│ │   │ ├─V-core
│ │   │ │ │   SINE with a 3' end derived from a
│ │   │ │ │   CR1-type LINE.
│ │   │ │ │
│ │   │ │ ├─CR1-end
│ │   │ │ │     SINE with a 3' end derived from a
│ │   │ │ │     CR1-type LINE.
│ │   │ │ │
│ │   │ │ ├─L2-end
│ │   │ │ ├─RTE-end
│ │   │ │ └─Unknown_LINE-dependent
│ │   │ └─V_and_MIR-core
│ │   │   │   SINE with a 3' end derived from a
│ │   │   │   L2-type LINE.
│ │   │   │
│ │   │   └─L2-end
│ │   │         SINE with a 3' end derived from a
│ │   │         L2-type LINE.
│ │   │
│ │   ├─tRNA_and_5S_RNA
│ │   │ │   SINEs lacking a central region or with
│ │   │ │   central region yet unrelated to that of
│ │   │ │   other SINEs.
│ │   │ │
│ │   │ └─No_or_Unknown_Core
│ │   │   │   SINEs lacking a central region or with
│ │   │   │   central region yet unrelated to that of
│ │   │   │   other SINEs.
│ │   │   │
│ │   │   └─Unknown_LINE-dependent
│ │   └─tRNA_and_7SL_RNA
│ │     │   SINEs lacking a central region or with
│ │     │   central region yet unrelated to that of
│ │     │   other SINEs.
│ │     │
│ │     ├─No-core
│ │     │ │   A retroposon mobilized by an L1-type
│ │     │ │   LINE.
│ │     │ │
│ │     │ └─L1-dependent
│ │     │       A retroposon mobilized by an L1-type
│ │     │       LINE.
│ │     │
│ │     └─No_or_Unknown_Core
│ │       │   SINEs lacking a central region or with
│ │       │   central region yet unrelated to that of
│ │       │   other SINEs.
│ │       │
│ │       └─Unknown_LINE-dependent
│ └─Retrotransposon
│   │   A placeholder for not-yet-classified
│   │   non-autonomous LTR elements with short
│   │   internal sequences.
```

```
│ │     │
│ │     ├─Long_Terminal_Repeat_Element
│ │     │ │   A placeholder for not-yet-classified
│ │     │ │   non-autonomous LTR elements with short
│ │     │ │   internal sequences.
│ │     │ │
│ │     │ ├─Bel-Pao
│ │     │ │     The third large group of LTR elements
│ │     │ │     besides Gypsy/ERV and Copia.
│ │     │ │
│ │     │ ├─Gypsy-ERV
│ │     │ │ │   A so far non-avian reptile specific
│ │     │ │ │   class of ERVs.
│ │     │ │ │
│ │     │ │ ├─Gypsy
│ │     │ │ ├─Pararetroviridae
│ │     │ │ │ │   Plant and insect DNA reverse-
│ │     │ │ │ │   transcribing viruses lacking a provirus
│ │     │ │ │ │   state in their cycle.
│ │     │ │ │ │
│ │     │ │ │ └─Caulimoviridae
│ │     │ │ │       Plant and insect DNA reverse-
│ │     │ │ │       transcribing viruses lacking a provirus
│ │     │ │ │       state in their cycle.
│ │     │ │ │
│ │     │ │ └─Retroviridae
│ │     │ │   │   A so far non-avian reptile specific
│ │     │ │   │   class of ERVs.
│ │     │ │   │
│ │     │ │   ├─Orthoretrovirinae
│ │     │ │   │ │   A so far non-avian reptile specific
│ │     │ │   │ │   class of ERVs.
│ │     │ │   │ │
│ │     │ │   │ ├─ERV1
│ │     │ │   │ │     Exogenous representatives are called
│ │     │ │   │ │     Gamma- and Epsilonretroviruses.
│ │     │ │   │ │
│ │     │ │   │ └─ERV2-group
│ │     │ │   │   │   A so far non-avian reptile specific
│ │     │ │   │   │   class of ERVs.
│ │     │ │   │   │
│ │     │ │   │   ├─ERV2
│ │     │ │   │   │     Exogenous representatives are called
│ │     │ │   │   │     Alpha-, Beta- and Delta-viruses.
│ │     │ │   │   │
│ │     │ │   │   ├─ERV3
│ │     │ │   │   │ │   Mammalian-specific non-autonomous LTR
│ │     │ │   │   │ │   elements dependent on class 3 (ERVL)
│ │     │ │   │   │ │   ERVs .
│ │     │ │   │   │ │
│ │     │ │   │   │ └─MaLR
│ │     │ │   │   │       Mammalian-specific non-autonomous LTR
│ │     │ │   │   │       elements dependent on class 3 (ERVL)
│ │     │ │   │   │       ERVs .
│ │     │ │   │   │
│ │     │ │   │   ├─ERV4
│ │     │ │   │   │     A so far non-avian reptile specific
│ │     │ │   │   │     class of ERVs.
│ │     │ │   │   │
│ │     │ │   │   └─Lenti
│ │     │ │   └─Spumaretrovirinae
│ │     │ ├─TRIM
│ │     │ │     A placeholder for not-yet-classified
│ │     │ │     non-autonomous LTR elements with short
│ │     │ │     internal sequences.
```

```
│ │     │ │
│ │     │ └─Ty1-Copia
│ │     ├─Penelope-like_Elements
│ │     └─Tyrosine_Recombinase_Elements
│ │       ├─DIRS
│ │       │ └─Q
│ │       ├─Ngaro
│ │       └─Viper-group
│ │         ├─TATE
│ │         └─Viper
│ └─Unknown
│   │   Primarily or only found in centromeric
│   │   regions.
│   │
│   └─Centromeric
│         Primarily or only found in centromeric
│         regions.
│
├─Low_Complexity
│     Stretches of sequence with unusually
│     simple composition. I.e. AT rich
│     sequence.
│
├─Other
│     A placeholder for unknown classes of
│     repetitive sequence.
│
├─Segmental_Duplication
│     Long segments of DNA with near-identical
│     sequence.
│
└─Tandem_Repeat
  │   Short pattern tandem repeats found in
  │   short runs.
  │
  ├─Satellite
  │ │   Primarily or only found in centromeric
  │ │   regions.
  │ │
  │ ├─Acromeric
  │ │     Satellites occurring specifically in the
  │ │     short arm of acromeric chromosomes.
  │ │
  │ ├─Centromeric
  │ │     Primarily or only found in centromeric
  │ │     regions.
  │ │
  │ ├─Macro
  │ ├─Subtelomeric
  │ ├─W-chromosomal
  │ └─Y-chromosomal
  └─Simple
        Short pattern tandem repeats found in
        short runs.
```
