# Supplementary material for: The Dfam community resource of transposable element families, sequence models, and genome annotations
Source: Mob DNA. 2021 Jan 12;12:2. doi: 10.1186/s13100-020-00230-y (PMC7805219; doi:10.1186/s13100-020-00230-y)
Supplement: Supplementary file 2 — Additional file 2. [file 13100_2020_230_MOESM2_ESM.pdf]

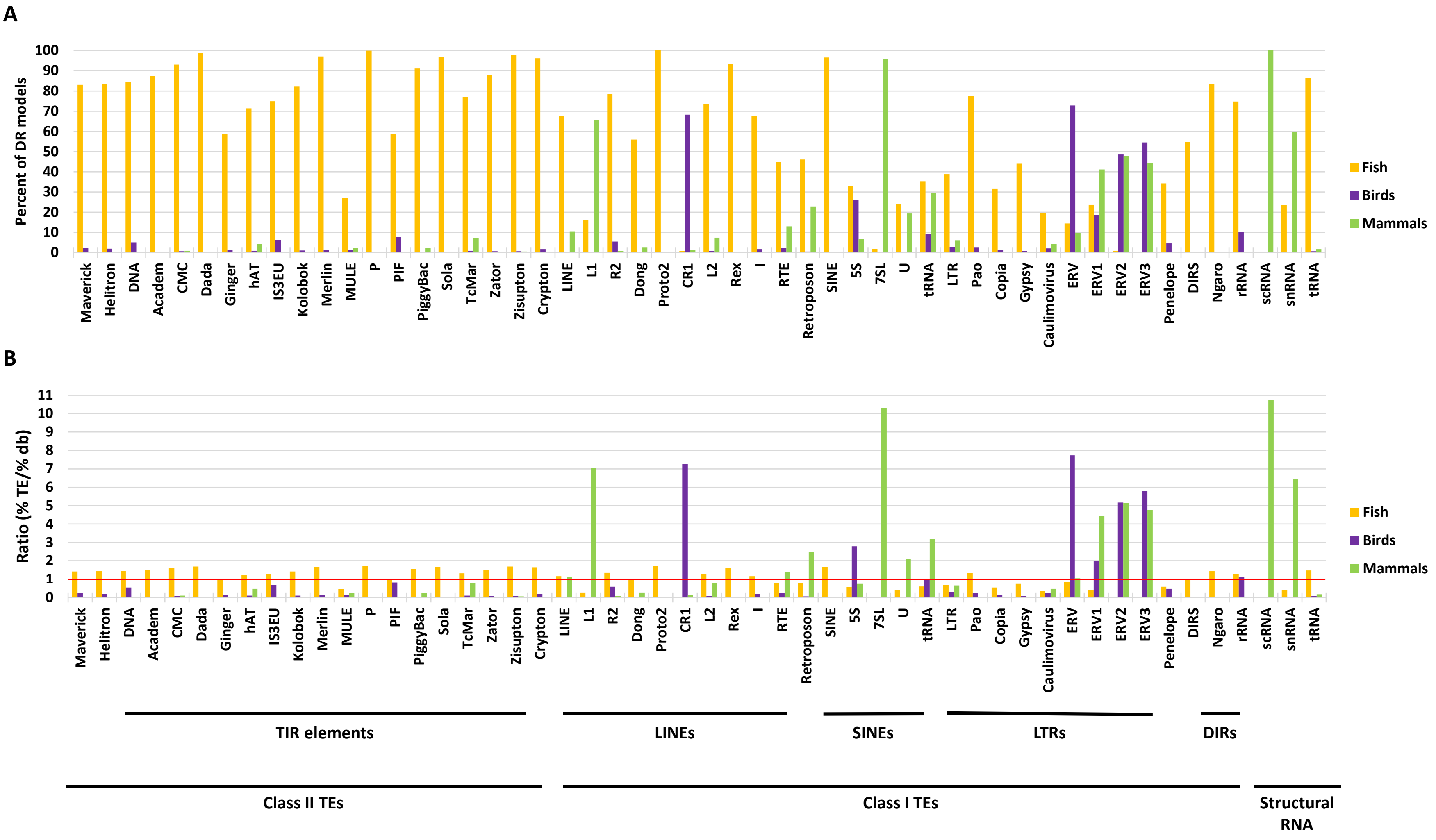

Supplemental Figure 2. EBI dataset TE class and superfamily contribution and enrichment by clade. (A) The percentage of TE classes and superfamilies observed for the fish, birds, and mammals TE models that comprise the total imported EBI TE models.(B) The ratio of the TE percentage taken from (A) divided by the percent of each of the clades' total family contribution to the EBI dataset. Any TE category above 1 (red line) is enriched for that TE type, while any TE category below 1 is depleted for that TE type.
