# Supplementary material for: The Dfam community resource of transposable element families, sequence models, and genome annotations
Source: Mob DNA. 2021 Jan 12;12:2. doi: 10.1186/s13100-020-00230-y (PMC7805219; doi:10.1186/s13100-020-00230-y)
Supplement: Supplementary file 4 — Additional file 4: Table S2. EBI dataset TE class and superfamily contribution and enrichment by clade for Supplemental Figure 2. [file 13100_2020_230_MOESM4_ESM.pdf]

Supplemental Table 2. EBI dataset TE class and superfamily contribution and enrichment by clade for Supplemental Figure 2.

Category	Subclass	RepeatMasker classification	All species #s	Fish	Birds	Mammals
			class II TEs			
DNA-polymerase	Polinton/Maverick	DNA/Maverick	265	220	6	0
Helicase	Heltiron	RC/Helitron	3654	3056	72	3
Transposase	Undefined	DNA	7379	6233	373	15
Transposase	Academ	DNA/Academ	221	193	0	1
Transposase	CACTA	DNA/CMC	3394	3157	24	34
Transposase	Dada	DNA/Dada	77	76	0	0
Transposase	Ginger	DNA/Ginger	68	40	1	0
Transposase	hAT	DNA/hAT	50396	35965	503	2211
Transposase	IS3EU	DNA/IS3EU	1388	1040	89	0
Transposase	Kolobok	DNA/Kolobok	2965	2436	32	0
Transposase	Merlin	DNA/Merlin	972	944	15	0
Transposase	MULE	DNA/MULE	748	202	9	17
Transposase	P element	DNA/P	1446	1444	1	0
Transposase	PIF/Harbinger	DNA/PIF	13412	7868	1029	3
Transposase	PiggyBac	DNA/PiggyBac	1685	1534	6	39
Transposase	Sola	DNA/Sola	584	565	0	1
Transposase	Tc1-Mariner	DNA/TcMar	19808	15274	181	1450
Transposase	Zator	DNA/Zator	133	117	1	0
Transposase	Zisupton	DNA/Zisupton	725	708	5	4
Y-recombinase	Crypton	DNA/Crypton	1362	1310	24	1
	**Total**		**110682**	**82382 (74.4%)**	**2371 (2.1%)**	**3779 (3.4%)**
			class I TEs			
LINE	Undefined	LINE	218	147	1	23
LINE	L1	LINE/L1	10717	1740	12	7016
LINE	R2	LINE/R2	380	298	21	3
LINE	R4/Dong	LINE/Dong	683	382	0	17
LINE	Proto2	LINE/Proto2	100	100	0	0
LINE	CR1	LINE/CR1	13073	107	8918	169
LINE	L2	LINE/L2	13220	9725	118	980
LINE	Rex/Babar	LINE/Rex	6478	6065	0	0
LINE	I	LINE/I	910	614	16	1
LINE	RTE/BovB	LINE/RTE	4608	2066	102	599
LINE-dependent	Long	Retroposon	328	151	2	75
LINE-dependent	Short (SINE)	SINE	471	455	1	0
LINE-dependent	5S-SINE	SINE/5S	423	140	111	29
LINE-dependent	7SL-SINE	SINE/7SL	569	11	0	545
LINE-dependent	U-SINE	SINE/U	62	15	0	12
LINE-dependent	tRNA-SINE	SINE/tRNA	5579	1971	514	1647
LTR	Undefined	LTR	1260	490	37	77
LTR	Bel-Pao	LTR/Pao	526	407	13	1
LTR	Ty1/Copia	LTR/Copia	1273	402	19	4
LTR	Ty3/Gypsy	LTR/Gypsy	10380	4567	87	36
LTR	Caulimoviruses	LTR/Caulimovirus	46	9	1	2
LTR	ERV-like	LTR/ERV	338	49	246	33
LTR	ERV1	LTR/ERV1	7671	1815	1435	3154
LTR	ERV2	LTR/ERV2	2974	30	1445	1425
LTR	ERV3	LTR/ERV3	8088	11	4407	3582
LTR	ERV4	LTR/ERV4	348	0	0	0
Penelope	Peneolope	Penelope	3536	1213	161	2
DIRS-like	DIRS	LTR/DIRS	2473	1353	1	1
DIRS-like	Ngaro	LTR/Ngaro	1452	1210	3	2
	**Total**		**98184**	**35534 (36.2%)**	**17770 (19.9%)**	**19433 (19.8%)**
structural RNA copies	rRNA	rRNA	194	145	20	0
structural RNA copies	scRNA	scRNA	7	0	0	7
structural RNA copies	snRNA	snRNA	174	41	0	104
structural RNA copies	tRNA	tRNA	597	516	4	10
	Satellite	Satellite	1054	540	275	64
	Unknown	Unknown	55421	36272	4555	1184
		Other	427	448	112	66
	**Total**		**266740**	**155887 (58.4%)**	**25108 (9.4%)**	**24649 (9.3%)**
